# Comparative Efficacy of Rebamipide, Pantoprazole, and Their Combination for Gastroprotection in Patients with Atrial Fibrillation Receiving Direct Oral Anticoagulants: The Randomized REGATA Study

**DOI:** 10.3390/medsci14050565

**Published:** 2026-09-12

**Authors:** Natalya M. Vorobyeva, Irina P. Malaya, Vadim D. Zakiev, Antonina A. Masharova, Tatiana P. Pinchuk, Irina V. Kostomarova, Valentina S. Ostapenko, Yulia V. Kotovskaya, Olga N. Tkacheva

**Affiliations:** Russian Gerontology Clinical and Research Center, Pirogov Russian National Research Medical University, 1st Leonova Street, 16, 129226 Moscow, Russia

**Keywords:** atrial fibrillation, direct oral anticoagulants, rebamipide, pantoprazole, gastroprotection, enteroprotection, mucosal permeability, fecal calprotectin, fecal zonulin

## Abstract

**Background:** Patients with atrial fibrillation (AF) receiving direct oral anticoagulants (DOAC) are at an elevated risk of gastrointestinal (GI) mucosal damage and bleeding. Proton pump inhibitors (PPI) are conventional gastroprotectors, but their efficacy in the lower GI tract is limited. This study evaluated the efficacy and safety of rebamipide monotherapy and combination pantoprazole-rebamipide (P + R) therapy compared to pantoprazole monotherapy in this vulnerable population. **Methods:** In the randomized, parallel-group, open-label, single-center REGATA trial, 210 AF patients receiving DOAC were randomized (1:1:1) to three gastroprotective regimens (*n* = 70 each) for 24 weeks: pantoprazole monotherapy, rebamipide monotherapy, or their combination. Efficacy analyses were performed in the per-protocol population (*n* = 118: pantoprazole, *n* = 36; rebamipide, *n* = 39; P + R, *n* = 43). The primary and secondary outcomes included composite clinical GI events (bleeding classified by ISTH/BARC, ulcers/erosions, and severe dyspepsia) and biomarkers of mucosal barrier function (fecal zonulin and calprotectin). **Results:** The incidence of primary efficacy endpoint events was 13.9% (*n* = 5; 95% CI: 3.1–25.7) in the pantoprazole group, 10.3% (*n* = 4; 95% CI: 2.3–21.1) in the rebamipide group, and 9.3% (*n* = 4; 95% CI: 2.1–18.6) in the P + R group, successfully confirming the non-inferiority hypothesis for both experimental arms within the pre-specified margin of 10% (*p* < 0.05). Multivariable regression analysis showed that compared with pantoprazole monotherapy, rebamipide monotherapy demonstrated an odds ratio OR of 0.71 (95% CI: 0.18–2.88, *p* = 0.630), while the P + R therapy was associated with an OR of 0.64 (95% CI: 0.16–2.57, *p* = 0.525). No major GI bleeding events or fatalities occurred; a single episode of GI bleeding (ISTH minor/BARC Type 2) was recorded in the pantoprazole arm (2.8%). Fecal calprotectin levels significantly decreased in the rebamipide group (by 37.6%, from 139 to 78 µg/g; *p* < 0.001) and the P + R group (by 50.0%, from 135 to 61 µg/g; *p* < 0.001), while no significant changes were observed in the pantoprazole group (*p* = 0.530). Concurrently, fecal zonulin levels, reflecting intestinal epithelial permeability, fell by 45.1% in the rebamipide arm (from 434 to 225 ng/mL; *p* < 0.001) and by 47.1% in the P + R arm (from 347 to 182 ng/mL; *p* < 0.001) compared to a minimal 3.0% fluctuation in the pantoprazole group. **Conclusions:** While demonstrating non-inferiority regarding primary clinical endpoints, both rebamipide monotherapy and combination (P + R) therapy achieved some advantages over pantoprazole monotherapy in decreasing subclinical GI inflammation and restoring intestinal epithelial barrier integrity.

## 1. Introduction

Atrial fibrillation (AF) is a highly prevalent cardiac arrhythmia associated with a substantial risk of cardioembolic stroke. Direct oral anticoagulants (DOAC) have demonstrated high efficacy in stroke prevention; however, their clinical utility is constrained by an elevated risk of hemorrhagic complications, particularly gastrointestinal bleeding (GIB), which can be fatal in some instances. Large-scale meta-analyses indicate that DOAC use is associated with a 25–45% increase in the risk of major GIB compared to warfarin [1,2]. Furthermore, an analysis of the US CDC WONDER database revealed that between 1999 and 2020, GIB-related mortality among AF patients more than doubled, from 0.55 to 1.16 per 100,000 individuals [3]. This upward trend may be partially attributed to the introduction and subsequent widespread adoption of DOAC in routine clinical practice.

Unlike vitamin K antagonists, which induce hemorrhagic complications predominantly through systemic anticoagulation, DOAC provoke GIB via both systemic and topical mechanisms [4]. The systemic effect is determined by the inhibition of coagulation factors IIa or Xa, which impairs adequate hemostasis at sites of subclinical epithelial lesions (such as erosions or angiodysplasias), thereby converting them into sources of active hemorrhage [4]. Conversely, the topical mechanism occurs when a significant fraction of the DOAC dose, particularly dabigatran etexilate and rivaroxaban, remains unabsorbed in the upper gastrointestinal (GI) tract and retains biological activity within the intestinal lumen [4]. Direct contact between the active drug and enterocytes disrupts intercellular tight junctions, increases mucosal permeability, inhibits epithelial repair, and exerts direct cytotoxicity [4]. Notably, the tartaric acid core in dabigatran capsules generates a localized acidic microenvironment that induces mucosal injury. Given the mandatory long-term nature of DOAC therapy, identifying effective gastroprotective strategies to mitigate GIB remains of paramount clinical importance.

Proton pump inhibitors (PPI) are traditionally used for gastroprotection in AF patients. Their mechanism of action relies on the inhibition of gastric parietal cell H^+^/K^+^-ATPase, thereby suppressing hydrochloric acid secretion [5]. As a result, PPI are effective primarily in preventing upper GIB and may conversely predispose patients to lower GIB [6,7,8]. Indeed, a meta-analysis of 12 studies involving 341,063 participants demonstrated that PPI use was associated with a more than threefold increase in the risk of lower GIB (hazard ratio [HR] 3.23; 95% confidence interval [CI] 1.56–6.71) [9]. Furthermore, recent data from a large population-based cohort study of 283,771 new DOAC users (mean age: 78.3 years, 49.4% female) indicated that, compared to DOAC monotherapy, concurrent DOAC and PPI therapy was associated with a higher cumulative incidence of adverse outcomes, including thrombotic events, bleeding, and all-cause mortality (20.2 vs. 15.2 per 100 patient-years, respectively) [10]. A secondary analysis of patients receiving this combination (*n* = 115,493) further revealed that periods of concomitant DOAC and PPI utilization were associated with a more than twofold increase in the risk of all-cause death (HR 2.24; 95% CI: 2.14–2.35) [10].

Consequently, effective protection of the GI mucosa necessitates a pharmacological agent with a mechanism of action distinct from that of PPI. Rebamipide, a gastro- and enteroprotector, fulfills this role by inducing the synthesis of endogenous prostaglandins, thereby restoring the structural integrity of the GI mucosal barrier across all its physiological levels. The existence of a topical mechanism underlying DOAC-induced GIB provides a strong pathogenetic rationale for the therapeutic deployment of rebamipide. Unlike PPI, which act indirectly by reducing the acidity of gastric secretions, rebamipide directly stimulates the synthesis of endogenous prostaglandins (PGE_2_ and PGI_2_) and glycosaminoglycans. This cascade reinforces the pre-epithelial and epithelial barriers and accelerates cell proliferation, effectively neutralizing the adverse topical effects of DOAC on the GI mucosa. According to a recent umbrella review of 11 meta-analyses comprising 88 primary studies [11], rebamipide showed significant efficacy in improving *H. pylori* eradication (odds ratio [OR] 1.76; 95% CI: 1.44–2.16) and reducing NSAID-induced mucosal injury (OR 2.72; 95% CI: 1.89–5.14). Furthermore, it enhanced ulcer healing following endoscopic submucosal dissection (ESD) (OR 2.28; 95% CI: 1.42–3.65) and effectively alleviated dyspeptic symptoms (OR 2.95; 95% CI: 1.04–8.37). However, despite these robust theoretical prerequisites, comparative clinical data regarding its efficacy as a monotherapy or in combination with other regimens in AF patients undergoing long-term DOAC therapy remain sparse.

This study aimed to investigate the comparative efficacy of three gastroprotective strategies (PPI monotherapy, rebamipide monotherapy, and their combination) in preventing GIB and associated adverse GI events in AF patients receiving DOAC.

## 2. Materials and Methods

### 2.1. Study Design and Patients

The REGATA study was designed as a single-center, open-label, prospective, randomized, controlled, parallel-group trial. The study was registered on ClinicalTrials.gov (Identifier: NCT07354230). The study was approved by the by the Local Ethics Committee of the RGCRC (protocol No. 48, dated 16 November 2021), and this study was performein accordance with the ethical standards as laid down in the 1964 Declaration of Helsinki and its later amendments or comparable ethical standards.

The study included patients aged ≥18 years with documented AF and a moderate-to-high risk of thromboembolic complications, defined as a CHA_2_DS_2_-VASc score ≥ 1 for men and ≥2 for women. Additionally, participants were required to be either currently receiving or prescribed stable DOAC therapy, and have clinical indications for gastroprotective therapy (rebamipide and/or pantoprazole).

Exclusion criteria comprised: (1) participation in other clinical trials within the previous 3 months; (2) contraindications to protocol-specified procedures; (3) pregnancy or lactation; and (4) active peptic ulcer disease or erosive lesions of the gastroduodenal mucosa at the time of screening.

**Figure 1:**
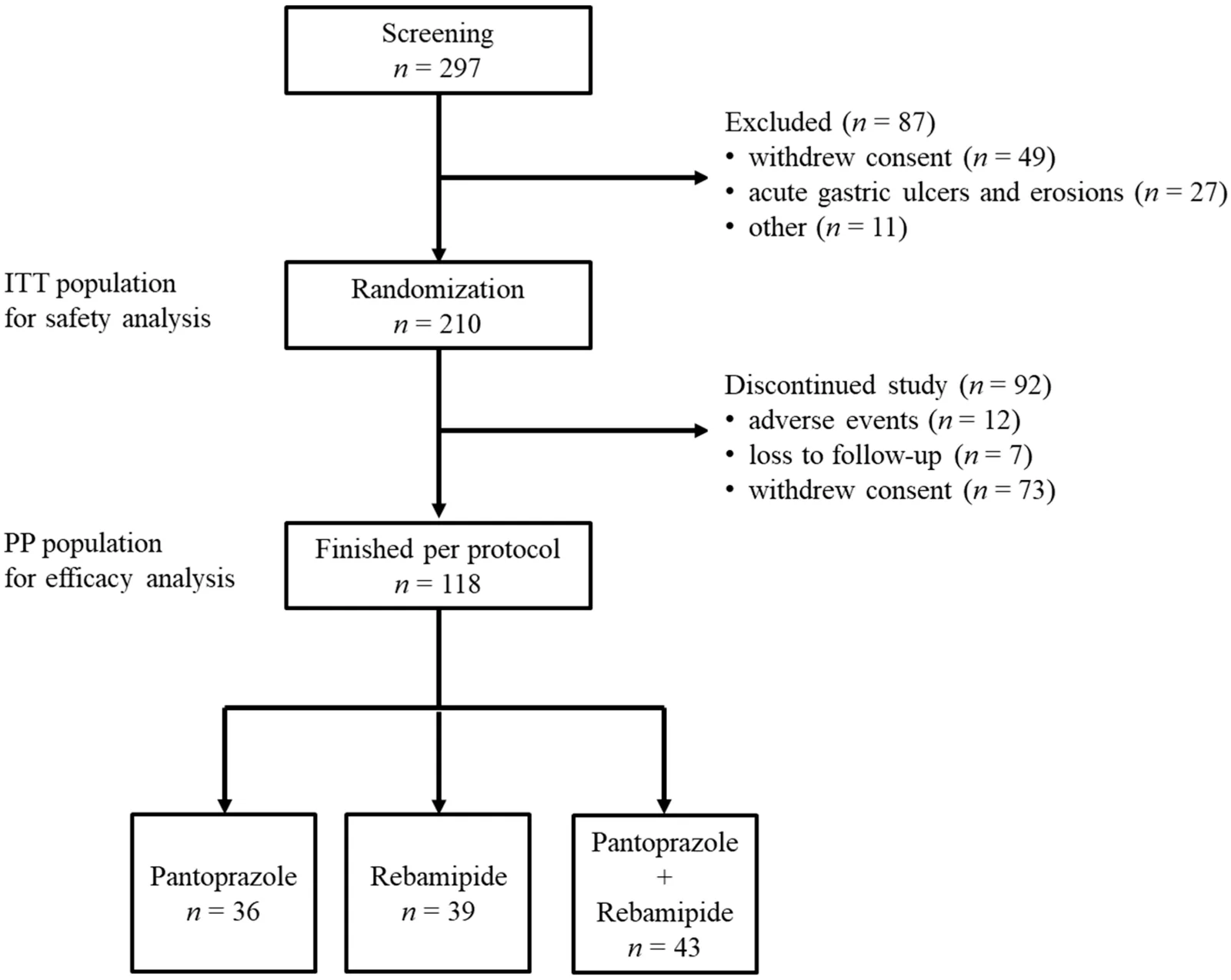
CONSORT flow diagram of patient screening, randomization, and treatment allocation. *Note.* ITT, intention-to-treat; PP, per protocol.

Between January 2022 and March 2024, a total of 297 patients underwent screening at the cardiology and internal medicine departments of the Russian Gerontology Clinical Research Center (Figure 1). Following the screening phase, 87 individuals were excluded, and 210 patients were enrolled. Participants were randomly assigned in a 1:1:1 ratio to three equal groups (*n* = 70 each) using a computer-generated randomization sequence: (1) pantoprazole monotherapy (40 mg once daily); (2) rebamipide monotherapy (100 mg three times daily); and (3) combination pantoprazole-rebamipide (P + R) therapy (pantoprazole 40 mg once daily plus rebamipide 100 mg three times daily). The therapeutic intervention lasted 24 weeks.

### 2.2. Study Procedures

The clinical phase of the study comprised a screening period (Visit 0), randomization (Visit 1), follow-up (Visits 2 and 3), and study completion (Visit 4).

Visits 0, 1, and 4 were conducted in an inpatient setting. Visits 1 and 4 (at 24 weeks) included the assessment of GI symptoms, evaluation of dyspepsia severity using the Global Overall Symptom (GOS) scale, and esophagogastroduodenoscopy (EGD) to grade the gastric and duodenal mucosa according to the Lanza scale. Additionally, *H. pylori* status was determined in biopsy specimens. Laboratory assessments included fecal occult blood test (FOBT) and measurements of fecal zonulin (a marker of increased epithelial permeability) and fecal calprotectin (a marker of inflammation).

Follow-up Visits 2 (at 8 weeks) and 3 (at 16 weeks) were conducted on an outpatient basis. Safety assessment, including the monitoring of concomitant therapy, adverse events (AE), and endpoint events, was performed at all follow-up visits (Visits 2, 3, and 4).

### 2.3. Diagnostic Assessments

Dyspepsia severity was assessed using the GOS scale [12].

EGD was performed according to standard protocols using an Olympus GIF-HQ-190 video gastroscope (Olympus Corp., Tokyo, Japan). Endoscopists performing the EGD procedures were blinded to the treatment allocation to ensure an unbiased assessment of mucosal lesions. During EGD, the gastric and duodenal mucosa were evaluated according to the Lanza scale [13]: 0, no hemorrhages, erosions, or ulcers; 1, hemorrhages only (<10); 2, 10–25 hemorrhages and/or 1–5 erosions; 3, >25 hemorrhages and/or 6–10 erosions; 4, >10 erosions or ulcer(s).

*H. pylori* infection was diagnosed using a rapid urease test (RUT) performed on gastric mucosal biopsy specimens obtained during EGD. In 18 patients (8.6%), RUT was not performed during endoscopy due to technical reasons; in these cases, *H. pylori* status was determined by detecting *H. pylori* antigen in stool samples via immunochromatography.

FOBT was conducted using a qualitative immunochromatographic method. Fecal calprotectin levels were measured via immunofluorescence assay, with normal values defined as <50 μg/g for individuals aged <65 years and <100 μg/g for those aged ≥65 years. Fecal zonulin levels were determined by enzyme-linked immunosorbent assay (ELISA) using IDK^®^ Zonulin ELISA kits (Immundiagnostik AG, Bensheim, Germany). The reference normal value for fecal zonulin was <83 ng/mL.

High-risk criteria for GIB were defined as follows: (1) a history of peptic ulcer disease or GIB; (2) chronic use of nonsteroidal anti-inflammatory drugs (NSAID) or corticosteroids; or (3) the presence of at least two of the following: age ≥ 65 years, dyspepsia, gastroesophageal reflux, *H. pylori* infection, or chronic alcohol consumption [14].

### 2.4. Primary and Secondary Endpoints

The primary endpoint was a composite of GI clinical and endoscopic events, defined as: (1) GIB classified according to the ISTH and BARC criteria; (2) gastric/duodenal ulcer or the presence of ≥5 gastroduodenal erosions; (3) GI obstruction or perforation; and (4) severe dyspepsia (GOS score 6–7).

The secondary endpoints included: (1) the individual components of the primary composite endpoint; (2) changes in dyspepsia severity based on the GOS score; (3) changes in the endoscopic appearance of the GI mucosa according to the Lanza scale; and (4) changes in FOBT positivity, as well as fecal zonulin and fecal calprotectin levels from baseline to week 24.

### 2.5. Statistical Analysis

Statistical analysis was performed using SPSS software (version 23.0; IBM Corp., Armonk, NY, USA). The sample size calculation was based on the primary non-inferiority design. Assuming an expected baseline incidence of the primary endpoint of 11% in the pantoprazole monotherapy group and 16% in both the rebamipide monotherapy and combination therapy groups, an absolute non-inferiority margin of 10% was defined. With a statistical power of 80% and a significance level of 0.05, a minimum of 64 participants per group was required to demonstrate non-inferiority. To account for a potential drop-out rate of approximately 10%, the target enrollment was set at 70 patients per treatment arm, resulting in a total sample size of 210 participants. Power and sample size analysis was performed using the Sealed Envelope power calculator for binary outcomes in non-inferiority trials (Sealed Envelope Ltd., London, UK).

Continuous data were analyzed using non-parametric statistical methods. Continuous and ordinal variables are presented as Med (Q_1_; Q_3_), where Med is the median, Q_1_ is the 1st quartile, and Q_3_ is the 3rd quartile. Categorical variables are expressed as absolute (*n*) and relative (%) frequencies. To assess intragroup longitudinal changes from baseline to week 24, McNemar’s test was applied for categorical variables, whereas the Wilcoxon signed-rank test was used for continuous and ordinal data. Pairwise intergroup comparisons for continuous and ordinal parameters were performed using the Mann-Whitney U test. Categorical variables were compared across groups using the two-sided Fisher’s exact test, and pairwise multiple comparisons for the Lanza scale score were adjusted using the Bonferroni correction. The 95% CIs for primary and secondary endpoint rates were calculated using the exact Clopper-Pearson method.

Because this study investigated pre-specified, planned pairwise comparisons of interest (the rebamipide monotherapy group vs. the pantoprazole group, and the combination therapy group vs. the pantoprazole group), no adjustments for multiple comparisons were applied. The primary non-inferiority hypothesis was tested by calculating the standard normal Z-statistic and the corresponding one-sided *p*-value based on the pre-specified non-inferiority margin. Incidence rates for clinical events were calculated and expressed as the number of events per 100 patient-years with their 95% CIs. Due to the small number of events, the 95% CIs were calculated using the exact Poisson method.

Per protocol (PP) and intention-to-treat (ITT) analyses were performed for the primary endpoint to minimize potential bias due to a substantial proportion of dropout patients. For the ITT population (*n* = 210), missing primary endpoint data for the 92 dropouts were handled via multiple imputation by fully conditional specification with 5 imputation datasets. The ITT multiple-imputation analysis was used strictly as a supportive sensitivity analysis. The primary non-inferiority outcomes were evaluated by calculating the absolute risk differences between the treatment arms along with their 95% CIs. To ensure strict mathematical consistency with the one-sided hypothesis testing (at an alpha level of 0.05) and to account for small-sample constraints, the primary non-inferiority criteria in the PP population were evaluated using the upper limit of the one-sided 95% CIs computed via the Farrington-Manning score method. For the supportive ITT analysis, the pooled absolute risk differences and their corresponding pooled two-sided 95% CIs were calculated using the traditional Wald estimation on each imputed dataset and subsequently combined using Rubin’s rules, based on the linear combination of within-imputation and between-imputation variances. Non-inferiority was formally established if the relevant upper confidence limits for the risk difference did not cross the prespecified non-inferiority margin of 10%.

To evaluate the independent impact of rebamipide monotherapy and combination therapy on the primary composite endpoint, a multivariable logistic regression analysis was performed, with pantoprazole monotherapy designated as the reference category. To ensure the robustness, stability, and internal validity of the regression model coefficients and 95% CIs, a bootstrap resampling technique (with 1000 replications) was integrated. All statistical tests were two-sided unless specified otherwise for the non-inferiority hypothesis, and a *p*-value of less than 0.05 was considered statistically significant.

## 3. Results

### 3.1. Participants

Of the 210 randomized patients, 118 (56.2%) completed the 24-week prospective follow-up PP, while 92 (43.8%) discontinued the study prematurely (Figure 1). The reasons for premature discontinuation included AE in 12 patients, loss to follow-up in 7, and withdrawal of consent in 73 (predominantly due to refusal to undergo follow-up EGD at Visit 4). The proportion of patients who completed the study PP was comparable across all three treatment arms. The total follow-up period accumulated 54.1 patient-years.

The baseline demographic, anthropometric, and clinical characteristics of the study cohort (*n* = 118) were well-balanced across the three treatment arms (Table 1). The cohort represents a typical vulnerable, elderly population (with medians ranging from 68 to 72 years) with a high cardiovascular and comorbidity burden. A key finding of this baseline analysis is the extraordinarily high prevalence of pre-existing GI disorders (75.0% to 84.6%) and a high risk of GIB (87.2% to 94.4%) among the participants.

### 3.2. Primary Endpoint

Regarding the primary efficacy endpoint, non-inferiority was successfully demonstrated for both the alternative gastroprotective monotherapy (rebamipide) and the intensified combination (P + R) strategy compared with the standard pantoprazole monotherapy. The incidence of primary efficacy endpoint events was 13.9% (*n* = 5; 95% CI: 3.1–25.7) in the pantoprazole group, 10.3% (*n* = 4; 95% CI: 2.3–21.1) in the rebamipide group, and 9.3% (*n* = 4; 95% CI: 2.1–18.6) in the combination (P + R) therapy group (Table 2). The overall incidence rate across the entire population was 11.0% (*n* = 13; 95% CI: 5.9–16.9).

The absolute risk difference for rebamipide monotherapy vs. pantoprazole was −3.6% (upper one-sided 95% confidence limit via Farrington-Manning: 9.2%), while the absolute risk difference for combination (P + R) therapy vs. pantoprazole was −4.6% (upper one-sided 95% confidence limit via Farrington-Manning: 7.5%). Because both upper confidence limits remained strictly below the pre-specified 10% non-inferiority margin, the null hypotheses of inferiority were successfully rejected for both the rebamipide monotherapy (*Z* = 2.42, *p*_non-inferiority_ = 0.008) and the combination (P + R) strategy (*Z* = 2.58, *p*_non-inferiority_ = 0.005).

To further evaluate the robustness of these findings against potential bias from missing data, a supportive sensitivity analysis was performed using multiple imputation with 5 imputation datasets across the ITT population. Following data pooling via Rubin’s rules using standard linear combinations of within- and between-imputation variances, the pooled absolute risk difference for rebamipide monotherapy vs. pantoprazole was −3.4% (pooled two-sided 95% CI: −16.3% to 9.4%), while the pooled absolute risk difference for the combination (P + R) therapy vs. pantoprazole was −4.6% (pooled two-sided 95% CI: −16.7% to 7.6%). Crucially, because the upper limits of both pooled intervals (9.4% and 7.6%, respectively) remained strictly below the pre-specified 10% non-inferiority threshold, the sensitivity analysis further reinforced the primary PP results, demonstrating high statistical stability and consistency of the trial’s conclusions across both populations.

A multivariable binary logistic regression model was utilized to evaluate the independent impact of the gastroprotective strategies on the risk of the primary composite endpoint. The global model did not reveal a statistically significant overall effect of the treatment groups (*χ*^2^ = 0.450, *df* = 2, *p* = 0.798). Specifically, compared with pantoprazole monotherapy, the alternative monotherapy with rebamipide demonstrated an OR of 0.71 (95% CI: 0.18–2.88, *p* = 0.630), while the combination (P + R) therapy was associated with an OR of 0.64 (95% CI: 0.16–2.57, *p* = 0.525). These regression findings were confirmed as robust by bootstrap internal validation (1000 replications). The overall incidence rate of primary endpoint events across the entire PP population was 24.0 per 100 patient-years (95% CI: 12.8–41.1).

### 3.3. Secondary Endpoints

The incidence of secondary efficacy endpoints is summarized in Table 2. Pairwise analysis revealed no statistically significant differences in these rates across the treatment arms.

**a. Gastrointestinal bleeding**

During the study period, no major GIB events or fatalities were reported. Only one episode of overt GIB occurred in a patient within the pantoprazole group, which was caused by an acute gastric ulcer. According to the pre-specified criteria, this event was classified as a minor bleeding episode per the ISTH definition and as a Type 2 bleeding event according to the BARC classification. The incidence of minor GIB was 0.8% (*n* = 1; 95% CI: 0.0–2.5) across the entire PP population and 2.8% (*n* = 1; 95% CI: 0.0–9.5) specifically within the pantoprazole monotherapy group. The overall incidence rate of minor GIB within the PP cohort was 1.9 per 100 patient-years (95% CI: 0.1–10.3).

**b. Gastric/duodenal erosion or ulcer**

The incidence of acute gastric/duodenal erosion or ulcer was 9.3% (*n* = 11; 95% CI: 4.2–15.3) across the entire PP population, 11.1% (*n* = 4; 95% CI: 2.6–22.2) in the pantoprazole group, 10.3% (*n* = 4; 95% CI: 2.1–20.0) in the rebamipide group, and 7.0% (*n* = 3; 95% CI: 0.0–16.0) in the combination therapy group (Table 2). The overall incidence rate of acute gastric/duodenal erosion or ulcer across the entire PP population was 20.3 per 100 patient-years (95% CI: 10.2–36.4).

Since the presence of acute gastric or duodenal erosions and ulcers served as an exclusion criterion, the baseline prevalence of drug-induced mucosal lesions across the entire cohort was low (hemorrhages: 10.2%; chronic erosion: 14.4%; chronic ulcers: 0.8%) and did not differ significantly between the study groups (Table 3). Despite the development of acute erosions and/or ulcers in 11 patients during the follow-up, the post-treatment frequencies of erosions and ulcers at week 24 showed no statistically significant differences among the three treatment arms (all *p* > 0.05). Notably, a statistically significant reduction in the prevalence of hemorrhages from baseline to week 24 was observed in the combination (P + R) group (from 14.0% to 0%, *p* = 0.031).

**c. Severe dyspepsia**

Two episodes of severe dyspepsia were reported (one in the pantoprazole group and one in the combination therapy group).

**d. Evolution of GI symptoms**

**d.1. Monotherapy Outcomes**

After 24 weeks of treatment, the pantoprazole monotherapy group demonstrated a significant reduction in upper GI symptoms, with the prevalence decreasing by 36.7% (from 83.3% to 52.8%; *p* = 0.003) (Table 4). This improvement was driven primarily by a 71.4% reduction in heartburn frequency (*p* = 0.021). However, pantoprazole monotherapy failed to provide significant relief for lower GI symptoms (*p* = 0.687), with high rates of abdominal bloating (61.1%) and flatulence (41.7%) persisting at week 24.

In the rebamipide group, the overall frequency of GI symptoms fell by 48.5% (from 84.6% to 43.6%, *p* < 0.001). Specifically, upper GI symptoms decreased by 61.3% (from 79.5% to 30.8%; *p* < 0.001) due to significant reductions in belching (by 77.8%; *p* = 0.016), epigastric heaviness (by 60.0%; *p* = 0.004), and epigastric discomfort (by 72.2%; *p* < 0.001). Additionally, lower GI symptoms in this group decreased by 54.1% (from 61.5% to 28.2%; *p* = 0.001), driven by a decline in abdominal bloating (by 69.7%; *p* = 0.001) and flatulence (by 75.0%; *p* = 0.022).

**d.2. Combination Therapy Outcomes**

In the combination (P + R) group, the overall frequency of GI symptoms fell by 55.9% (from 79.1% to 34.9%; *p* < 0.001) (Table 4). Upper GI symptoms decreased by 69.7% (from 76.7% to 23.3%; *p* < 0.001), characterized by an 87.5% reduction in heartburn (*p* = 0.001), an 88.9% decline in epigastric heaviness (*p* < 0.001), and an 81.2% drop in epigastric discomfort (*p* < 0.001). Notably, complete resolution at week 24 was achieved for both belching (100% reduction, from 16.3% to 0.0%; *p* = 0.016) and epigastric pain (100% reduction, from 18.6% to 0.0%; *p* = 0.008).

Additionally, lower GI symptoms decreased by 64.3% (from 65.1% to 23.3%; *p* < 0.001), driven by prominent reductions in abdominal bloating (by 82.6%; *p* < 0.001), flatulence (by 81.2%; *p* < 0.001), and abdominal rumbling (by 77.8%; *p* = 0.016).

**d.3. Intergroup Comparisons**

Intergroup analysis at week 24 revealed significant clinical advantages of the combination therapy over pantoprazole monotherapy (Table 4). The combination (P + R) regimen demonstrated superior efficacy in reducing the overall frequency of GI symptoms compared to pantoprazole alone (34.9% vs. 72.2%; *p* = 0.001), as well as a significantly lower residual prevalence of upper GI symptoms (23.3% vs. 52.8%; *p* = 0.010). This clear advantage of the combination therapy group was driven by a significantly lower rate of epigastric heaviness (4.7% vs. 22.2%; *p* = 0.037), epigastric discomfort (7.0% vs. 27.8%; *p* = 0.016), and the complete absence of belching (0.0% vs. 11.1%; *p* = 0.039) compared to the pantoprazole group.

Furthermore, both rebamipide-containing regimens (monotherapy and combination) achieved substantially better control over lower GI symptoms than pantoprazole. At week 24, lower GI symptoms were significantly less frequent in the rebamipide group (28.2%; *p* < 0.001) and the P + R group (23.3%; *p* < 0.001) than in the pantoprazole group (69.4%). This difference was primarily due to a lower prevalence of abdominal bloating (15.4% and 9.3% vs. 61.1%; *p* < 0.001 for both comparisons) and flatulence (7.7% and 7.0% vs. 41.7%; *p* = 0.001 and *p* < 0.001, respectively).

**e. Evolution of dyspepsia severity by GOS scale**

After 24 weeks of treatment, all three groups demonstrated a significant intra-group reduction in symptom severity (Table 5). However, both the rebamipide monotherapy and the combination (P + R) therapy groups achieved a substantially lower median GOS score compared to the pantoprazole monotherapy group (1 [1; 2] vs. 2 [1.25; 3]; *p* < 0.001 for both comparisons). The relative reduction in GOS scores from baseline was significantly more pronounced in the rebamipide-containing regimens (−50.0%) than in the pantoprazole group (−12.5%; *p* = 0.004 for rebamipide vs. pantoprazole, and *p* < 0.001 for P + R vs. pantoprazole).

**f. Evolution of mucosal damage by Lanza score**

Regarding mucosal damage assessed by the Lanza scale, the overall median scores remained at 0 (0; 0) across all groups at both time points due to a substantial floor effect, and no significant intra-group changes were observed (*p* > 0.05) (Table 5). However, categorical stratification of mucosal lesions revealed significant treatment disparities at week 24. A statistically significant overall advantage was found for the combination (P + R) therapy group over the pantoprazole monotherapy group (*p* = 0.046). This benefit was primarily driven by a higher rate of complete mucosal healing (Lanza score = 0) in the P + R cohort compared to the pantoprazole cohort (88.4% vs. 69.4%, respectively; *p* = 0.050). In contrast, the prevalence of lesion-free mucosa remained stagnant in the pantoprazole group (69.4% at both baseline and week 24).

**g. Evolution of fecal calprotectin**

Fecal calprotectin levels were elevated at baseline in a high proportion of patients across all groups, with no significant baseline differences (*p* > 0.05) (Table 5). By week 24, fecal calprotectin concentrations remained virtually unchanged in the pantoprazole group (183 [80; 216] vs. 174 [93; 251] µg/g at baseline; *p* = 0.530). In contrast, calprotectin levels dropped significantly in both the rebamipide group (from 139 [55; 225] to 78 [40; 101] µg/g; *p* < 0.001) and the combination (P + R) therapy group (from 135 [79; 188] to 61 [42; 96] µg/g; *p* < 0.001). The relative reduction from baseline was significantly more pronounced in the rebamipide (−37.6%) and P + R (−50.0%) regimens compared to the minimal fluctuation in the pantoprazole cohort (0.9%; *p* = 0.002 for rebamipide vs. pantoprazole, and *p* < 0.001 for P + R vs. pantoprazole). Intergroup analysis at week 24 confirmed that post-treatment calprotectin levels were substantially lower in both rebamipide-containing groups than in the pantoprazole cohort (*p* < 0.001 for both comparisons). Consequently, the proportion of patients with elevated calprotectin levels decreased significantly only in the rebamipide monotherapy group (from 66.7% to 30.8%; *p* < 0.001) and the P + R combination group (from 74.4% to 37.2%; *p* < 0.001).

**h. Evolution of fecal zonulin**

A highly consistent pattern was observed for fecal zonulin levels, a key proxy for intestinal epithelial barrier permeability (Table 5). While pantoprazole monotherapy led to a minimal 3.0% decrease in zonulin levels (from 531 [267; 643] to 468 [207; 581] ng/mL; *p* = 0.042), rebamipide monotherapy and P + R therapy mediated robust reductions of 45.1% (from 434 [266; 765] to 225 [120; 443] ng/mL; *p* < 0.001) and 47.1% (from 347 [173; 488] to 182 [81; 303] ng/mL; *p* < 0.001), respectively. At week 24, post-treatment zonulin levels were significantly lower in the rebamipide (225 ng/mL; *p* = 0.028) and P + R groups (182 ng/mL; *p* = 0.002) compared to the pantoprazole group (468 ng/mL). Notably, combination therapy was the only regimen that achieved a statistically significant reduction in the proportion of patients with elevated baseline zonulin levels by week 24 (from 93.0% to 74.4%; *p* = 0.039), whereas this parameter remained unchanged in the pantoprazole (*p* = 0.625) and rebamipide (*p* = 1.000) monotherapy arms.

**i. Evolution of FOBT positivity**

At baseline and at week 24, the prevalence of positive FOBT was comparable across all three groups (Table 5). Although no statistically significant longitudinal changes were observed within the groups, the positive FOBT rate numerically shifted from 16.7% to 27.8% in the pantoprazole group (*p* = 0.289), from 17.9% to 15.4% in the rebamipide monotherapy group (*p* = 1.000), and from 14.0% to 11.6% in the combination therapy group (*p* = 1.000).

Thus, while demonstrating comparable efficacy to pantoprazole monotherapy regarding primary endpoint events, both rebamipide-containing regimens exhibited distinct advantages. These regimens significantly reduced the frequency and severity of dyspeptic symptoms, while successfully lowering the levels of fecal calprotectin, a marker of intestinal inflammation, and fecal zonulin, a key proxy for intestinal epithelial barrier permeability.

### 3.4. Safety Analysis

AEs assessments were conducted in the intention-to-treat population. Over the 24-week gastroprotective therapy period, a total of 64 AEs were recorded among 58 patients (Table 6). No statistically significant differences were observed among the three groups. All documented AEs were of mild-to-moderate intensity. Due to AE development, 12 patients permanently discontinued their assigned study medications and withdrew prematurely from the trial: 3 in the rebamipide group, 7 in the pantoprazole group, and 2 in the combination (P + R) therapy group. The causal relationship between study drug administration and the AE was classified as “probable” for these 12 cases of premature discontinuation. For all other reported AE, the relationship to the study treatment was deemed “unlikely.” The overall rate of premature study discontinuation driven by AE was 5.7% (12/210; 95% CI: 2.9–8.6). Crucially, no serious AE or treatment-related deaths occurred during the study.

## 4. Discussion

This paper presents the results of the REGATA study, a randomized, controlled, prospective trial involving 210 AF patients who received DOAC (dabigatran, rivaroxaban, or apixaban) alongside various gastroprotective regimens (rebamipide, pantoprazole, or their combination) for 24 weeks. At baseline and week 24, all patients underwent EGD with mucosal injury assessed via the Lanza scale, *H. pylori* infection testing, dyspepsia symptom severity evaluation using the GOS scale, FOBT, and measurements of fecal calprotectin and zonulin levels. To our knowledge, this is the first study utilizing such a design and diagnostic methodology in AF patients managed with DOAC.

Of the 210 randomized patients, 118 (56.2%) completed the study in accordance with the protocol. A total of 92 (43.8%) patients prematurely discontinued the study: 12 due to AE, 7 were lost to follow-up, and 73 withdrew consent, primarily due to refusing a follow-up EGD at Visit 4. The study protocol required two hospitalizations (at baseline and 24 weeks) to undergo EGD. At baseline, chronic erosive or ulcerative gastroduodenal lesions were detected in only 15.3% of patients (acute lesions served as exclusion criteria). Consequently, given the initial absence of erosions and ulcers, many patients refused the follow-up EGD, particularly those without ulcer history and whose GI symptoms had either resolved or significantly diminished.

Premature patient discontinuation, including an unwillingness to undergo specific study procedures, is a well-documented phenomenon and not unique to the REGATA trial. For instance, a randomized trial published in 2021 [15] evaluated 204 patients undergoing ESD for early gastric cancer or adenoma. Given that ESD results in substantial mucosal defects with a high risk of GIB, this trial compared the efficacy of three gastroprotective regimens for postoperative ulcer healing and GIB prevention: (1) PPI monotherapy; (2) PPI plus rebamipide; and (3) an H_2_-receptor antagonist (H2RA) plus rebamipide. A follow-up EGD to assess therapeutic efficacy was scheduled one month post-ESD, meaning each patient was subjected to the endoscopic procedures twice within a 1-month period. Following randomization, 48 individuals withdrew from the trial: 26 withdrew informed consent, and 22 did not undergo EGD for unspecified reasons. Consequently, only 156 patients were included in the final analysis, yielding a 24% withdrawal rate, reflecting a similar behavioral trend to that observed in the REGATA trial. This demonstrates that even oncology patients facing a tangible threat of postoperative GIB frequently decline follow-up EGD. Notably, the combination of a PPI and rebamipide demonstrated the highest efficacy for postoperative ulcer healing, whereas rebamipide monotherapy was not evaluated. Postoperative GIB incidence was 0% in the PPI group, 3.85% (*n* = 2) in the PPI + rebamipide group, and 7.7% (*n* = 4) in the H2RA + rebamipide group (*p* = 0.126).

Although the high attrition rate is the primary limitation of the REGATA study, the trial yielded statistically significant data demonstrating the efficacy and safety of the evaluated gastroprotective regimens. The 24-week gastroprotective therapy effectively prevented adverse GI events. No episodes of major GIB, GI obstruction, or perforation occurred, and the incidence of acute erosions and ulcers remained below 10% with no statistically significant differences between the treatment groups. During the follow-up period, only one patient (in the pantoprazole group) experienced a GIB episode from an acute gastric ulcer, which was classified as minor. This case illustrates that while PPI do not provide absolute protection against GIB, they may mitigate bleeding severity by enhancing thrombus stabilization via gastric acid suppression [16]. Notably, no GIB events occurred in patients receiving rebamipide monotherapy or the rebamipide-pantoprazole combination.

In the REGATA study, the 24-week incidence of GIB was 0.8% (1.9 per 100 patient-years), which is substantially lower than rates reported in literature. For instance, a retrospective cohort study from Hong Kong [6] involving 5041 patients treated with dabigatran (mean age: 72 years; 48% male; AF prevalence: 88%) reported a GIB incidence of 2.5% (4.2 per 100 patient-years). Concomitant gastroprotective therapy with dabigatran was associated with a 48% reduction in GIB risk (HR 0.52; 95% CI: 0.35–0.77) compared to patients with no gastroprotection. Specifically, PPI therapy decreased GIB risk by 47% (HR 0.53; 95% CI: 0.31–0.91), whereas H2RA reduced it by 39% (HR 0.61; 95% CI: 0.40–0.94). Further analysis revealed that gastroprotective therapy exclusively reduced the risk of upper GIB by 71% (HR 0.29; 95% CI: 0.15–0.54). Moreover, this protective effect was most pronounced in patients with a history of peptic ulcer disease or prior GIB, demonstrating an 86% risk reduction (HR 0.14; 95% CI: 0.06–0.30).

A Japanese retrospective cohort study [7] evaluated 658 patients (mean age: 72 years; 68% male) receiving DOAC (dabigatran, *n* = 220; rivaroxaban, *n* = 283; apixaban, *n* = 155). The overall incidence of GIB was 2.0% per year, with major GIB accounting for 0.9% per year. Notably, this study demonstrated a higher incidence of lower GIB compared to upper GIB (1.3% vs. 0.7% per year, respectively).

Another retrospective cohort study [8] evaluated the efficacy of gastroprotective agents in mitigating upper GIB risk among 2076 patients receiving DOAC, of whom only 360 (17%) received gastroprotection. The incidence of upper GIB was 0.3% (0.7 per 100 patient-years) in the gastroprotection cohort and 1.7% (2.8 per 100 patient-years) in patients without gastroprotective therapy (*p* = 0.189), indicating that the use of gastroprotective agents did not significantly reduce the risk of upper GIB.

Data from the Russian REGATTA registry [17] enrolled 510 AF patients (median age: 67 years; 60% male), all of whom initially received warfarin, with 44% subsequently switched to DOAC. Over a 20-year follow-up period, 32 major GIB events occurred (22 during warfarin therapy and 10 during DOAC therapy), translating to a major GIB incidence of 1.42 per 100 patient-years. Since the REGATTA registry reflects real-world clinical practice, the potential role of gastroprotective therapy in GIB prevention was not investigated. Only 37% of the patients received intermittent PPI therapy.

Consequently, the incidence of GIB across all mentioned studies was higher than that observed in the REGATA trial, which recorded no major GIB events. Furthermore, three out of the four studies utilized a retrospective design, and none evaluated the efficacy of rebamipide. However, it is critical to note that since only a single episode of minor GIB was registered in our study, we are unable to definitively evaluate the comparative impact of the investigated gastroprotective regimens on GIB risk.

The REGATA study revealed a distinct enteroprotective effect of rebamipide. Although the enteroprotective properties of this agent are well-established, they have been primarily demonstrated in experimental models or in patients receiving NSAID or acetylsalicylic acid (ASA) for short durations (typically 2–4 weeks), with the gastroprotective regimen matching the period of NSAID or ASA administration. In most previous trials, rebamipide was compared with a placebo; however, selected studies indicated that its efficacy is non-inferior or equivalent to that of PPI, H2RA, and misoprostol. Specifically, a meta-analysis of 15 randomized controlled trials (RCT) involving 965 patients [18] showed that rebamipide is more effective than a placebo for healing erosions and ulcers in NSAID-induced enteropathy (OR 2.70; 95% CI: 1.02–7.16; *p* = 0.045). The REGATA trial confirmed the enteroprotective effect of rebamipide in AF patients receiving DOAC over a 24-week gastroprotective treatment period.

Evidence supporting the enteroprotective effect of rebamipide includes a substantial reduction in the incidence of lower GI symptoms (both overall symptoms and specific complaints such as abdominal bloating, flatulence, and borborygmi) and a significant decrease in the levels of fecal calprotectin and zonulin.

Importantly, fecal calprotectin and zonulin levels in AF patients receiving DOAC remain virtually unexplored. Apart from the REGATA trial, fecal calprotectin has been evaluated in only one study [19], which involved 166 AF patients undergoing radiofrequency catheter ablation. In that trial, calprotectin was measured within 1–7 days after ablation to assess its diagnostic utility as a marker of ablation-induced esophageal injury. Two other studies evaluated circulating zonulin levels in AF patients. In the first [20], serum zonulin was assessed in 80 patients (72.5% aged ≥ 65 years; 62.5% male) with newly diagnosed AF and compared with 20 healthy controls. In the second study [21], serum zonulin levels were determined in 100 patients (median age: 69 years; 58% male) undergoing elective cardiac surgery with cardiopulmonary bypass, 38% of whom developed postoperative AF.

A key limitation of the aforementioned studies is the cross-sectional assessment of intestinal biomarkers at a single time point. Consequently, longitudinal data evaluating the dynamics of fecal calprotectin and zonulin in AF patients receiving DOAC and gastroprotective therapy remain scarce. Therefore, the findings obtained from the REGATA study provide novel insights into this clinical context. Another methodological advantage of this study lies in the simultaneous evaluation of both fecal calprotectin and zonulin levels within the same patient cohort.

To date, only two studies have evaluated the dynamics of fecal calprotectin and zonulin levels during rebamipide therapy, neither of which involved AF patients. These trials differed substantially from the REGATA study, primarily in terms of patient cohort characteristics and treatment duration.

In 2016, a Japanese RCT evaluated the protective effects and optimal dosing of rebamipide against gastrointestinal mucosal injury induced by low-dose ASA [22]. The trial enrolled 45 healthy volunteers who received 100 mg/day enteric-coated ASA for 14 days. They were randomized to receive concomitant therapy with either omeprazole 10 mg/day, rebamipide 300 mg/day or rebamipide 900 mg/day. Upper endoscopy, capsule endoscopy, fecal occult blood testing, and fecal calprotectin measurements were performed at baseline and post-treatment. Following the 14-day intervention, fecal calprotectin levels increased significantly in the omeprazole group (from 2.67 ± 4.25 to 22.19 ± 32.48 ng/g; *p* = 0.004), whereas no significant changes were observed in the 300 mg/day rebamipide group (from 5.79 ± 8.32 to 7.78 ± 14.75 ng/g; *p* = 0.844) or the 900 mg/day rebamipide group (from 5.88 ± 11.17 to 2.48 ± 4.20 ng/g; *p* = 0.438). These findings suggest that rebamipide attenuates fecal calprotectin elevation, potentially via its anti-inflammatory properties. Furthermore, the standard dosage (300 mg/day) appeared sufficient to prevent low-dose ASA-induced small intestinal mucosal injury, demonstrating no additional therapeutic advantage with the higher dose (900 mg/day). In contrast to the REGATA study, that trial evaluated calprotectin dynamics in healthy volunteers rather than AF patients on DOAC, and the treatment duration was limited to 14 days.

The evolution of serum zonulin (in contrast to fecal zonulin in the REGATA study) during rebamipide therapy were evaluated in a Russian RCT involving 60 patients with diarrhea-predominant irritable bowel syndrome (IBS-D) [23]. The trial assessed symptom severity, intestinal barrier integrity, and gut microbiota composition and function. Patients were randomized to receive trimebutine, rebamipide, or their combination for 2 months. Rebamipide monotherapy significantly improved intestinal barrier function, mitigated enterocyte injury, and attenuated inflammatory responses. Notably, a significant reduction in serum zonulin was observed exclusively in the rebamipide monotherapy cohort. The median change in zonulin levels was 4.6 (interquartile range [IQR]: −6.3 to 11.7) ng/mL in the trimebutine group, −1.3 (IQR: −3.8 to 0.0) ng/mL in the combination group, and −12.3 (IQR: −16.9 to −4.7) ng/mL in the rebamipide group (*p* = 0.006 vs. trimebutine; *p* = 0.003 vs. combination therapy). These data indicate that rebamipide monotherapy was superior to trimebutine, whereas combining both agents yielded no additional benefit. Unlike the REGATA study, this trial focused on serum zonulin dynamics in patients with IBS-D over a 2-month treatment period.

The REGATA trial evaluated the 24-week longitudinal dynamics of fecal calprotectin and zonulin within a single cohort of AF patients receiving DOAC. To our knowledge, this is the first study to investigate these specific biomarker trajectories in this clinical setting. Although all three gastroprotective strategies demonstrated comparable overall efficacy, rebamipide monotherapy offered distinct advantages over pantoprazole by exerting enteroprotective effects and achieving a more pronounced reduction in dyspeptic symptoms. Furthermore, combining rebamipide with pantoprazole provided synergistic benefits, including robust enteroprotection, complete resolution of mucosal hemorrhage, and a reduction in gastric and duodenal erosive-ulcerative lesion severity according to the Lanza scale. This combination therapy also significantly decreased the incidence of GI symptoms involving both the upper and lower GI tracts and led to superior alleviation of dyspepsia.

In conclusion, the REGATA trial provides the first 24-week comparative evaluation of the efficacy and safety of three gastroprotective regimens (rebamipide, pantoprazole, and their combination) in AF patients on DOAC therapy.

### Study Limitations

Several limitations of the REGATA study should be acknowledged.

First, a significant limitation is the substantial drop-out rate observed over the 24-week follow-up period, with 92 out of 210 randomized participants (43.8%) failing to complete the study according to the protocol. While such attrition is not uncommon in longitudinal studies involving complex therapeutic regimens and elderly populations on chronic anticoagulants, it reduced the statistical power of the PP efficacy analyses. To rigorously address this issue and mitigate potential attrition bias, we performed an ITT analysis using multiple imputation for missing data. The consistency observed between the ITT and PP conclusions validates the robustness of our core findings.

Second, a formal, strict quantitative assessment of pill-intake compliance was not performed.

Third, this was a single-center trial conducted within a specific geographic population, which may restrict the external validity and generalizability of the results to broader, multi-ethnic patient cohorts.

Fourth, the open-label nature of the trial could theoretically introduce performance or detection bias, particularly regarding subjective patient-reported outcomes such as dyspepsia severity scores.

Finally, only a single episode of minor GIB was registered during the study. This low event rate prevents us from drawing definitive conclusions regarding the comparative impact of pantoprazole, rebamipide, or their combination on actual clinical bleeding risks or long-term gastrointestinal complications that might emerge over multi-year direct oral anticoagulant therapy.

## Figures and Tables

**Table 1:** Baseline characteristics of AF patients receiving DOAC (PP population, *n* = 118).

Parameter	Pantoprazole (1) *n* = 36	Rebamipide (2) *n* = 39	P + R (3) *n* = 43	*p*-Value (2–1)	*p*-Value (3–1)
**Demographic characteristics**					
Age, years	72 (66; 75)	72 (66; 76)	68 (61; 74)	0.602	0.185
Male sex	9 (25.0)	10 (25.6)	18 (41.9)	0.949	0.116
**Anthropometric characteristics**					
Body mass index, kg/m^2^	30.9 (26.6; 35.1)	29.9 (26.6; 34.2)	31.1 (25.9; 34.2)	0.675	0.933
Overweight	9 (25.0)	15 (38.5)	13 (30.2)	0.212	0.605
Obesity	22 (61.1)	19 (48.7)	23 (53.5)	0.281	0.496
**Clinical characteristics**					
AF pattern:				0.369	0.457
Paroxysmal	19 (52.8)	24 (61.5)	19 (44.2)		
Persistent	1 (2.8)	3 (7.7)	4 (9.3)		
Permanent	16 (44.4)	12 (30.8)	20 (46.5)		
Duration of AF, years	5 (2; 9)	5 (3; 12)	4 (2; 5)	0.404	0.890
History of radiofrequency ablation	2 (5.6)	7 (17.9)	3 (7.0)	0.156	1.000
History of electrical cardioversion	2 (5.6)	7 (17.9)	3 (7.0)	0.156	1.000
CHA_2_DS_2_-VASc score, points	4 (3; 6)	5 (3; 6)	4 (3; 5)	0.474	0.248
HAS-BLED score, points	1 (1; 2)	1 (1; 2)	1 (0; 2)	0.690	0.273
DOAC type:				0.924	0.670
Apixaban	21 (58.3)	24 (61.5)	21 (48.8)		
Dabigatran	1 (2.8)	2 (5.1)	2 (4.7)		
Rivaroxaban	14 (38.9)	13 (33.3)	20 (46.5)		
Concomitant antiplatelet/NSAID use	4 (11.1)	6 (15.4)	2 (4.7)	0.738	0.403
**Comorbidities**					
Hypertension	35 (97.2)	38 (97.4)	40 (93.0)	1.000	0.621
Coronary artery disease	8 (22.2)	8 (20.5)	9 (20.9)	0.857	0.889
History of myocardial infarction	8 (22.2)	6 (15.4)	6 (14.0)	0.448	0.338
Peripheral artery disease	4 (11.1)	4 (10.3)	6 (14.0)	1.000	0.748
History of ischemic stroke	5 (13.9)	8 (20.5)	2 (4.7)	0.449	0.236
History of hemorrhagic stroke	0 (0.0)	1 (2.6)	1 (2.3)	1.000	1.000
Chronic heart failure	25 (69.4)	31 (79.5)	32 (74.4)	0.318	0.623
Type 2 diabetes mellitus	11 (30.6)	9 (23.1)	13 (30.2)	0.464	0.975
Anemia	7 (19.4)	5 (12.8)	9 (20.9)	0.434	0.870
**Gastrointestinal disorders**					
Any gastrointestinal disease	27 (75.0)	33 (84.6)	35 (81.4)	0.298	0.491
Chronic gastritis	25 (69.4)	28 (71.8)	29 (67.4)	0.823	0.849
Gastroesophageal reflux disease	15 (41.7)	25 (64.1)	21 (48.8)	0.052	0.524
Gastric or duodenal ulcer disease	8 (22.2)	6 (15.4)	12 (27.9)	0.448	0.563
Hemorrhoids	2 (5.6)	6 (15.4)	3 (7.0)	0.265	1.000
History of endoscopic gastric/colorectal polypectomy	3 (8.3)	3 (7.7)	1 (2.3)	1.000	0.326
History of gastric surgery *	1 (2.8)	0 (0.0)	2 (4.7)	0.480	1.000
History of intestinal surgery **	0 (0.0)	1 (2.6)	3 (7.0)	1.000	0.246
Inflammatory bowel disease (UC or Crohn’s)	1 (2.8)	0 (0.0)	0 (0.0)	0.480	0.456
History of GI bleeding	1 (2.8)	0 (0.0)	1 (2.3)	0.480	1.000
High risk of GI bleeding	34 (94.4)	34 (87.2)	38 (88.4)	0.433	0.445
Positive fecal occult blood test	6 (16.7)	7 (17.9)	6 (14.0)	0.883	0.738
Concomitant anemia plus positive FOBT	4 (11.1)	2 (5.1)	2 (4.7)	0.419	0.403
*H. pylori* infection	25 (69.4)	20 (51.3)	27 (62.8)	0.157	0.535

Note. Data are presented as Med (Q_1_; Q_3_) or *n* (%). AF, atrial fibrillation; CHA_2_DS_2_-VASc, Congestive heart failure, Hypertension, Age ≥ 75 years (doubled), Diabetes mellitus, Stroke/transient ischemic attack/thromboembolism (doubled), Vascular disease, Age 65–74 years, Sex category (female); DOAC, direct oral anticoagulant; FOBT, fecal occult blood test; GI, gastrointestinal; HAS-BLED, Hypertension, Abnormal renal/liver function, Stroke, Bleeding history or predisposition, Labile international normalized ratio, Elderly (age > 65 years), Drugs/alcohol concomitantly; NSAID, nonsteroidal anti-inflammatory drug; PP, per protocol; P + R, Pantoprazole + Rebamipide; UC, ulcerative colitis; not calculated. * Includes perforated ulcer repair, gastric resection, gastrostomy, reconstructive and oncological surgeries. ** Includes bowel obstruction surgery, intestinal resection, colostomy, reconstructive and oncological surgeries.

**Table 2:** Incidence of primary and secondary endpoints during 24 weeks of gastroprotective therapy in AF patients receiving DOAC (PP population, *n* = 118).

Parameter	Overall	Pantoprazole (1)	Rebamipide (2)	P + R (3)	*p*-Value (2–1)	*p*-Value (3–1)
Randomized, *n*	210	70	70	70	-	-
Completed study per protocol	118 (56.2)	36 (51.4)	39 (55.7)	43 (61.4)	0.735	0.306
Primary efficacy endpoint *	13 (11.0)	5 (13.9)	4 (10.3)	4 (9.3)	0.730	0.724
**Secondary efficacy endpoints**						
GI bleeding:						
Major	0 (0.0)	0 (0.0)	0 (0.0)	0 (0.0)	n/c	n/c
Minor	1 (0.8)	1 (2.8)	0 (0.0)	0 (0.0)	0.480	0.456
Gastric/duodenal ulcer or ≥5 erosions	11 (9.3)	4 (11.1)	4 (10.3)	3 (7.0)	1.000	0.696
GI obstruction or perforation	0 (0.0)	0 (0.0)	0 (0.0)	0 (0.0)	n/c	n/c
Severe dyspepsia (GOS score 6–7)	2 (1.7)	1 (2.8)	0 (0.0)	1 (2.3)	0.480	1.000

Note. Data are presented as *n* (%). AF, atrial fibrillation; DOAC, direct oral anticoagulant; PP, per protocol; P + R, Pantoprazole + Rebamipide; GI, gastrointestinal; GOS, Global Overall Symptom; n/c, not calculated. * Includes GI bleeding, gastric/duodenal ulcer or the presence of ≥5 gastroduodenal erosions, GI obstruction or perforation, and severe dyspepsia (GOS score 6–7).

**Table 3:** Changes of endoscopic mucosal lesions during 24 weeks of gastroprotective therapy in AF patients receiving DOAC (PP population, *n* = 118).

Endoscopic Feature	Time Point/ Analysis	Pantoprazole (1) *n* = 36	Rebamipide (2) *n* = 39	P + R (3) *n* = 43	*p*-Value (2–1)	*p*-Value (3–1)
Hemorrhages	Baseline	4 (11.1)	2 (5.1)	6 (14.0)	0.419	0.748
	Week 24	2 (5.6)	1 (2.6)	0 (0.0)	0.605	0.204
	*p*-value (within group) *	0.625	1.000	**0.031**		
Erosions	Baseline	8 (22.2)	5 (12.8)	4 (9.3)	0.365	0.129
	Week 24	9 (25.0)	7 (17.9)	4 (9.3)	0.575	0.074
	*p*-value (within group) *	1.000	0.687	1.000		
Ulcers	Baseline	0 (0.0)	0 (0.0)	1 (2.3)	n/c	1.000
	Week 24	1 (2.8)	2 (5.1)	1 (2.3)	1.000	1.000
	*p*-value (within group) *	1.000	0.500	1.000		

Note. Data are presented as *n* (%). AF, atrial fibrillation; DOAC, direct oral anticoagulant; PP, per protocol; P + R, Pantoprazole + Rebamipide; n/c, not calculated. * Comparison between baseline and week 24 values within each treatment group. Bold is used to show the statistical significance.

**Table 4:** Changes in GI symptoms and intergroup comparisons at baseline and week 24 of gastroprotective therapy in AF patients receiving DOAC (PP population, *n* = 118).

Symptoms	Pantoprazole (1) *n* = 36			Rebamipide (2) *n* = 39			P + R (3) *n* = 43			*p*-Value (2–1)		*p*-Value (3–1)	
	Baseline	Week 24	*p*-Value	Baseline	Week 24	*p*-Value	Baseline	Week 24	*p*-Value	Baseline	Week 24	Baseline	Week 24
**Any GI symptoms**	31 (86.1)	26 (72.2)	0.063	33 (84.6)	17 (43.6)	**<0.001**	34 (79.1)	15 (34.9)	**<0.001**	1.000	**0.019**	0.557	**0.001**
**Upper GI symptoms**	30 (83.3)	19 (52.8)	**0.003**	31 (79.5)	12 (30.8)	**<0.001**	33 (76.7)	10 (23.3)	**<0.001**	0.771	0.064	0.578	**0.010**
Nausea	2 (5.6)	1 (2.8)	1.000	3 (7.7)	0 (0.0)	0.250	3 (7.0)	1 (2.3)	0.500	1.000	0.480	1.000	1.000
Heartburn	14 (38.9)	4 (11.1)	**0.021**	10 (25.6)	4 (10.3)	0.146	16 (37.2)	2 (4.7)	**0.001**	0.322	1.000	1.000	0.403
Belching	5 (13.9)	4 (11.1)	1.000	9 (23.1)	2 (5.1)	**0.016**	7 (16.3)	0 (0.0)	**0.016**	0.381	0.419	1.000	**0.039**
Epigastric pain	9 (25.0)	3 (8.3)	0.109	6 (15.4)	3 (7.7)	0.508	8 (18.6)	0 (0.0)	**0.008**	0.390	1.000	0.586	0.090
Epigastric heaviness	14 (38.9)	8 (22.2)	0.180	15 (38.5)	6 (15.4)	**0.004**	18 (41.9)	2 (4.7)	**<0.001**	1.000	0.557	0.822	**0.037**
Epigastric discomfort	11 (30.6)	10 (27.8)	1.000	18 (46.2)	5 (12.8)	**<0.001**	16 (37.2)	3 (7.0)	**<0.001**	0.236	0.150	0.636	**0.016**
Other	2 (5.6)	1 (2.8)	1.000	1 (2.6)	1 (2.6)	1.000	1 (2.3)	2 (4.7)	1.000	0.605	1.000	0.589	1.000
**Lower GI symptoms**	27 (75.0)	25 (69.4)	0.687	24 (61.5)	11 (28.2)	**0.001**	28 (65.1)	10 (23.3)	**<0.001**	0.228	**<0.001**	0.462	**<0.001**
Abdominal bloating	20 (55.6)	22 (61.1)	0.727	20 (51.3)	6 (15.4)	**0.001**	23 (53.5)	4 (9.3)	**<0.001**	0.818	**<0.001**	1.000	**<0.001**
Flatulence	10 (27.8)	15 (41.7)	0.063	12 (30.8)	3 (7.7)	**0.022**	16 (37.2)	3 (7.0)	**<0.001**	0.805	**0.001**	0.473	**<0.001**
Abdominal rumbling	6 (16.7)	6 (16.7)	1.000	7 (17.9)	3 (7.7)	0.289	9 (20.9)	2 (4.7)	**0.016**	1.000	0.298	0.775	0.132
Abdominal pain	3 (8.3)	2 (5.6)	1.000	3 (7.7)	1 (2.6)	0.625	3 (7.0)	2 (4.7)	1.000	1.000	0.605	1.000	1.000
Constipation	13 (36.1)	12 (33.3)	1.000	8 (20.5)	7 (17.9)	1.000	11 (25.6)	8 (18.6)	0.375	0.198	0.184	0.336	0.194
Diarrhea	0 (0.0)	2 (5.6)	0.500	1 (2.6)	1 (2.6)	1.000	5 (11.6)	0 (0.0)	0.063	1.000	0.605	0.059	0.204
Other	1 (2.8)	0 (0.0)	1.000	0 (0.0)	0 (0.0)	n/c	0 (0.0)	0 (0.0)	n/c	0.480	n/c	0.456	n/c

Note. Data are presented as *n* (%). AF, atrial fibrillation; DOAC, direct oral anticoagulant; PP, per protocol; P + R, Pantoprazole + Rebamipide; GI, gastrointestinal; n/c, not calculated. Bold is used to show the statistical significance.

**Table 5:** Changes in symptom scores, endoscopic mucosal lesions, FOBT positivity, and intestinal permeability biomarkers after 24 weeks of gastroprotective therapy in AF patients receiving DOAC (PP population, *n* = 118).

Parameter	Pantoprazole (1) *n* = 36	Rebamipide (2) *n* = 39	P + R (3) *n* = 43	*p*-Value (2–1)	*p*-Value (3–1)
**GOS scale, points**					
Baseline	3 (2; 4)	3 (2; 3)	3 (2; 4)	0.087	0.613
Week 24	2 (1.25; 3)	1 (1; 2)	1 (1; 2)	**<0.001**	**<0.001**
Change from baseline (%)	−12.5 (−50.0; −12.5)	−50.0 (−50.0; −20.0)	−50.0 (−56.2; −29.6)	**0.004**	**<0.001**
*p*-value (within group) *	**0.023**	**<0.001**	**<0.001**		
**Lanza scale, points**					
Baseline, Med (Q_1_; Q_3_)	0 (0; 2)	0 (0; 0)	0 (0; 0)	0.106	0.402
0, *n* (%)	25 (69.4)	33 (84.6)	33 (76.7)	0.236 **	0.805 **
1–2, *n* (%)	7 (19.4)	5 (12.8)	7 (16.3)		
3–4, *n* (%)	4 (11.1)	1 (2.6)	3 (7.0)		
Week 24, Med (Q_1_; Q_3_)	0 (0; 2)	0 (0; 0)	0 (0; 0)	0.396	**0.046**
0, *n* (%)	25 (69.4) ^a^	31 (79.5)	38 (88.4) ^a^	0.372 **	0.088 **
1–2, *n* (%)	7 (19.4) ^a^	3 (7.7)	2 (4.7) ^b^		
3–4, *n* (%)	4 (11.1) ^a^	5 (12.8)	3 (7.0) ^a^		
*p*-value (within group) *	0.762	0.150	0.586		
**Fecal calprotectin, µg/g**					
Baseline	174 (93; 251)	139 (55; 225)	135 (79; 188)	0.261	0.102
Week 24	183 (80; 216)	78 (40; 101)	61 (42; 96)	**<0.001**	**<0.001**
Change from baseline (%)	0.9 (−34.9; 14.4)	−37.6 (−57.3; −15.2)	−50.0 (−61.2; −31.3)	**0.002**	**<0.001**
*p*-value (within group) *	0.530	**<0.001**	**<0.001**		
Elevated level at baseline	28 (77.8)	26 (66.7)	32 (74.4)	0.284	0.728
Elevated level at week 24	26 (72.2)	12 (30.8)	16 (37.2)	**<0.001**	**0.002**
*p*-value (within group) *	0.727	**<0.001**	**<0.001**		
**Zonulin in stool, ng/mL**					
Baseline	531 (267; 643)	434 (266; 765)	347 (173; 488)	0.791	0.076
Week 24	468 (207; 581)	225 (120; 443)	182 (81; 303)	**0.028**	**0.002**
Change from baseline (%)	−3.0 (−27.3; 4.2)	−45.1 (−57.0; −29.3)	−47.1 (−56.2; −29.6)	**<0.001**	**<0.001**
*p*-value (within group) *	**0.042**	**<0.001**	**<0.001**		
Elevated level at baseline	34 (94.4)	36 (92.3)	40 (93.0)	0.711	1.000
Elevated level at week 24	32 (88.9)	35 (89.7)	32 (74.4)	1.000	0.102
*p*-value (within group) *	0.625	1.000	**0.039**		
**Positive fecal occult blood test**					
Baseline	6 (16.7)	7 (17.9)	6 (14.0)	0.883	0.738
Week 24	10 (27.8)	6 (15.4)	5 (11.6)	0.191	0.068
*p*-value (within group) *	0.289	1.000	1.000		

Note. Data are presented as Med (Q_1_; Q_3_) or *n* (%). AF, atrial fibrillation; DOAC, direct oral anticoagulant; PP, per protocol; P + R, Pantoprazole + Rebamipide; GOS, Global Overall Symptom. * Comparison between baseline and week 24 values within each treatment group. ** The *p*-value refers to the overall distribution of Lanza scale scores (0, 1–2, vs. 3–4 points) between the groups, analyzed using the Fisher’s exact test for 3 × 2 contingency tables. ^a,b^ Values in the same row crying different superscript letters differ significantly at the 0.05 level (adjusted via Bonferroni correction for multiple comparisons). Bold is used to show the statistical significance.

**Table 6:** Incidence and clinical spectrum of adverse events during 24 weeks of gastroprotective therapy in AF patients receiving DOAC (ITT population, *n* = 210).

Adverse Event	Pantoprazole (1) *n* = 70	Rebamipide (2) *n* = 70	P + R (3) *n* = 70	*p*-Value (2–1)	*p*-Value (3–1)
Nausea	0 (0.0)	3 (4.3)	1 (1.4) *	0.245	1.000
Heartburn	0 (0.0)	3 (4.3) *	0 (0.0)	0.245	n/c
Epigastric pain	0 (0.0)	1 (1.4)	0 (0.0)	1.000	n/c
Abdominal pain	1 (1.4) *	0 (0.0)	1 (1.4) *	1.000	1.000
Constipation	1 (1.4) *	2 (2.9)	1 (1.4)	1.000	1.000
Diarrhea	2 (2.9) *	0 (0.0)	0 (0.0)	0.496	0.496
Headache	1 (1.4)	0 (0.0)	1 (1.4)	1.000	1.000
Dizziness	0 (0.0)	1 (1.4)	0 (0.0)	1.000	n/c
Skin rash	2 (2.9) *	0 (0.0)	0 (0.0)	0.496	0.496
Pruritus	1 (1.4) *	0 (0.0)	0 (0.0)	1.000	1.000
Insomnia	0 (0.0)	1 (1.4)	1 (1.4)	1.000	1.000
Dry mouth	2 (2.9)	0 (0.0)	1 (1.4)	0.496	1.000
Pyrexia	0 (0.0)	0 (0.0)	1 (1.4)	n/c	1.000
Acute respiratory tract infection	9 (12.9)	7 (10.0)	6 (8.6)	0.791	0.586
Radial fracture	0 (0.0)	0 (0.0)	1 (1.4)	n/c	1.000
Somnolence	1 (1.4)	1 (1.4)	2 (2.9)	1.000	1.000
Nervousness	1 (1.4)	0 (0.0)	0 (0.0)	1.000	1.000
Increased appetite	2 (2.9)	1 (1.4)	1 (1.4)	1.000	1.000
Tinnitus	2 (2.9)	2 (2.9)	0 (0.0)	1.000	0.496

* Led to permanent study drug discontinuation. Note. Data are presented as *n* (%). AF, atrial fibrillation; DOAC, direct oral anticoagulant; ITT, intention-to-treat; P + R, Pantoprazole + Rebamipide. n/c, not calculated.

## Data Availability

The original contributions presented in this study are included in the article. Further inquiries can be directed to the corresponding authors.

## Abbreviations

AE	Adverse events
AF	Atrial fibrillation
ASA	Acetylsalicylic acid
BARC	Bleeding Academic Research Consortium
CHA_2_DS_2_-VASc	Congestive heart failure, Hypertension, Age ≥ 75 years, Diabetes mellitus, Stroke, Vascular disease, Age 65–74 years, Sex category (female)
CI	Confidence interval
DOAC	Direct oral anticoagulants
EGD	Esophagogastroduodenoscopy
ESD	Endoscopic submucosal dissection
FOBT	Fecal occult blood test
GI	Gastrointestinal
GIB	Gastrointestinal bleeding
GOS	Global Overall Symptom
HAS-BLED	Hypertension, Abnormal renal/liver function, Stroke, Bleeding history or predisposition, Labile INR, Elderly, Drugs/alcohol concomitantly
HR	Hazard ratio
H2RA	H_2_-receptor antagonist
IBS-D	Diarrhea-predominant irritable bowel syndrome
IQR	Interquartile range
ISTH	International Society on Thrombosis and Haemostasis
ITT	Intention-to-treat
Med	Median
NSAID	Nonsteroidal anti-inflammatory drugs
OR	Odds ratio
P	Pantoprazole
PGE_2_	Prostaglandin E_2_
PGI_2_	Prostaglandin I_2_
PP	Per protocol
PPI	Proton pump inhibitors
Q_1_	1st quartile
Q_3_	3rd quartile
R	Rebamipide
RCT	Randomized controlled trial
RUT	Rapid urease test
